# Mobile Monitoring and Reasoning Methods to Prevent Cardiovascular Diseases

**DOI:** 10.3390/s130506524

**Published:** 2013-05-16

**Authors:** Ramón Hervás, Jesús Fontecha, David Ausín, Federico Castanedo, Diego López-de-Ipiña, José Bravo

**Affiliations:** 1 MAmI Research Lab, University of Castilla-La Mancha, Esc. Sup. de Informática, Paseo de la Universidad, 4, 13071 Ciudad Real, Spain; E-Mails: jesus.fontecha@uclm.es (J.F.); jose.bravo@uclm.es (J.B.); 2 Deusto Institute of Technology, DeustoTech. University of Deusto, Avda. de las Universidades, 24, 48007 Bilbao, Spain; E-Mails: david.ausin@deusto.es (D.A.); fcastanedo@deusto.es (F.C.); dipina@deusto.es (D.L.-I.)

**Keywords:** mobile monitoring, ambient assisted living, CVD risk, blood pressure, reasoning

## Abstract

With the recent technological advances, it is possible to monitor vital signs using Bluetooth-enabled biometric mobile devices such as smartphones, tablets or electric wristbands. In this manuscript, we present a system to estimate the risk of cardiovascular diseases in Ambient Assisted Living environments. Cardiovascular disease risk is obtained from the monitoring of the blood pressure by means of mobile devices in combination with other clinical factors, and applying reasoning techniques based on the Systematic Coronary Risk Evaluation Project charts. We have developed an end-to-end software application for patients and physicians and a rule-based reasoning engine. We have also proposed a conceptual module to integrate recommendations to patients in their daily activities based on information proactively inferred through reasoning techniques and context-awareness. To evaluate the platform, we carried out usability experiments and performance benchmarks.

## 1. Introduction

The concept of ubiquitous computing in healthcare has become a reality. This is explained by the advent of new embedded technologies, the wide use of universally deployed devices, such as smartphones and tablets, and advances in wireless communications. The Ambient Assisted Living (AAL) initiative (http://www.aal-europe.eu/) promotes the adoption of information and communication technologies for helping elderly people, who live alone at home, to perform their daily activities. The goal is to increase end-user's quality of life but bearing in mind that it is crucial to serve users in terms of usability. In this sense, the continuous monitoring of vital signs is essential to determine the health condition of the person at any moment. This becomes especially important when a person suffers from a chronic disease or pathology that must be continuously checked. Many times, a continuous monitoring implies the use of biometric devices to obtain measures related to clinical parameters such as glucose or blood pressure among others. Other factors from the patient profile and/or personal medical record must also be taken into account, for example: age, sex, healthy habits, measures from analytical tests (e.g., cholesterol level) and even social factors (e.g., the country where the person lives). All of them have a specific importance, depending on the monitoring goal. In this paper, we will focus on blood pressure monitoring and several related factors to determine the total risk of suffering a Cardiovascular Disease (CVD) for a patient. For that, we have combined a reasoning engine based on Systematic Coronary Risk Evaluation Project (SCORE) chart [1] hosted on a server with a Bluetooth mobile monitoring software.

The rest of this article is organized as follows. The next section provides an overview of related AAL systems. Then Section 3 describes the architecture of the proposed system to estimate CVD risk. In Section 4 we describe the user evaluation and benchmark experiments. Finally Section 5 summarizes the article and points future work.

## 2. Related Work

### 2.1. Monitoring Systems

In the recent years, mobile technologies have been integrated in many AAL systems to improve and monitor several activities of daily living at users home. In our particular domain, Bravo *et al.* [2] propose a model to collect monitoring data by using mobile devices. On the one hand, the Health Buddy System project [3] connects patients at their homes with physicians to avoid the hospitalization; and the Health@Home project [4] presents a monitoring system for people affected by chronic heart failure. This system connects a home care system with the Hospital Information System and the patient is monitored through wireless sensors. In several projects, monitoring tasks are based on questionnaires and the physicians receive the results through internet. Moreover, this kind of systems requires to explicitly introduce the information by the users.

On the other hand, Bluetooth specifications (http://bluetooth.com) are being integrated in standard biometric devices, enabling many kinds of monitoring. In fact, the Continua Health Alliance (http://www.continuaalliance.org) promotes the use of a standard datasheet or protocol to receive and manage information from Bluetooth biometric devices.

The MoMo project [5] presents a framework based on several ontological models to facilitate the development of mobile monitoring systems integrating biometric and mobile devices.

### 2.2. Ontologies and Clinical Decision Support Systems

Clinical Decision Support Systems (CDSS) are conceived to automate and improve clinical decision making. Hunt *et al.* [6] have studied the effects of clinical decision support systems on physicians and patients and concluded that these systems may be useful. They pointed out that the benefits for diagnosis are not clear and patient outcomes should be studied in depth. A new update of this study [7] indicates that when using CDSS patient outcomes remain understudied and shows an improvement of clinical decision support systems for diagnosis tasks.

Description logics have been also employed in medical informatics for tasks [8] as terminology modeling (*i.e.*, OpenGALEN [9] and SNOMED CT [10]) or decision support. Description logics are the base of the OWL Web Ontology Language (OWL) [11]. OWL language has been successfully applied to model data and reason upon them in other domains as well. In OWL, there is a trade-off between the expressivity and the reasoning time: the more expressive an ontology is, the slower the reasoning task is. Another feature of the OWL ontology is that it can be combined with Semantic Web Rule Language (SWRL) [12], thus new knowledge about a given semantic model can not only be generated through the built-in ontological reasoning but also through expert-defined rules. Therefore, we propose the use of reasoning mechanisms taking into account OWL and SWRL features for the final CVD risk estimation.

Bodenreider [13] classifies biomedical ontologies into three categories: (i) knowledge management; (ii) data integration, exchange and semantic interoperability; (iii) and decision support and reasoning. They also point out that ontologies are available in different formats, such as RRF, OBO or OWL.

Abidi *et al.* presented [14] a breast cancer follow-up decision support system. This work combines their own OWL ontologies with Jena rules to assist family physicians in the breast cancer diagnosis and treatment.

HEARTFAID [15] has developed a clinical decision support system to detect heart failure. It describes the domain combining OWL ontologies with SWRL rules and SPARQL for querying the ontologies.

Farion *et al.* [16] proposed a client-server system, named Mobile Emergency Triage, to deal with heterogeneous clinical decision problems. In this case, authors used frame based representation to model the ontology.

A tool to facilitate antibiotic prescription was developed by Bright *et al.* [17]. They employed OWL and SWRL rules for generating alerts and providing feedback to the physicians in order to guide the antibiotic prescription.

## 3. Cardiovascular Disease Risk Estimation System

The aim of this work is to support clinical decisions and to enable the estimation of CVD risk and related recommendations from the monitoring of the blood pressure at home taking into account the clinical data of the user. The CVD risk is estimated applying the SCORE method. SCORE is currently the main methods employed by the European Societies of Cardiology to determine CVD risk percentage in European and Mediterranean countries. However, our work can be extrapolated to non-European regions turning their CVD standardized charts into SWRL rules to be used by our system depending on the specific region. For example, the Framingham Risk Score [18] is the most common method for CVD risk estimation in USA, but this is also used elsewhere in the world. Most CVD methods are based on clinical evidences and their results are similar because they have been created from a base chart score proposed by the World Health Organization (WHO) and the International Society of Hypertension (ISH) [19] but adjusted to different regions. We have chosen SCORE because the system is being developed and evaluated in Spain.

Our goals are achieved by using the MoMo framework principles combined with several clinical factors from the patient record. Collected data are represented using an adaptation of the MoMo ontology and are the input of a reasoning task based on OWL and SWRL rules.

### 3.1. Principles and Adaptation of MoMo Framework

The MoMo framework [5] allows the development of mobile applications in an adaptive, generic and remote way. *Generic* means enabling the development of applications for any kind of disease. *Adaptive* means providing services adjusted to each disease depending on the patient profile. *Remote* means that medical staff is able to access all the data gathered by the patient biometric devices in a non-intrusive manner. *Mobile* means allowing the development of applications based on the integration of small wireless devices. This framework proposes the use of design patterns to develop user interfaces and a standard modular system. It also describes an ontological classification called *MoMOntology* providing a data formalization, including patient profile, diseases and recommendations. In this work, we have used the previous principles and adapt the MoMo patient profile ontology with a set of SWRL rules to estimate CVD risk.

### 3.2. Blood Pressure Monitoring

**Figure 1.**
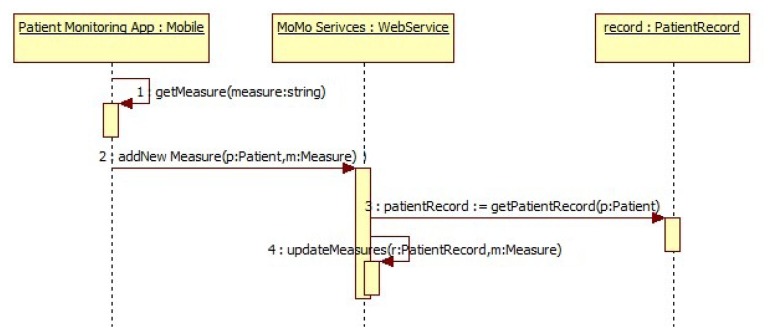
Sequence diagram: A new measure of blood pressure has been taken.

The European guidelines on CVD prevention in clinical practice [1] suggest checking the blood pressure levels frequently to prevent coronary diseases. In this sense, the daily frequency to measure the blood pressure depends on the health condition of the person and his patient record [20]. To get measures we have developed an Android mobile application connected to a Bluetooth-powered pressure monitor, namely Stabil O GRAPH SBPM-Control (http://www.iem.de/stabil_o_graph_mobil2). These measures are stored in a remote database by means of web services (explained in detail in Section 3.6). This mobile application promotes the autonomy of the user since the intervention of the physician to take new measures is not needed. In Figure 1 the sequence diagram for blood pressure monitoring is shown.

Most of the time, single measures do not provide sufficient information, therefore, more complex analysis are carried out. Thus, we must take into account other factors in order to make a proper assessment of risks. In the case of hypertensive people, the top 10 high blood pressure risk factors [21] include: age, ethnicity, gender, family history, smoking, activity level, diet, medication and street drugs, kidney problems, and other medical problems. A continuous blood pressure monitoring is also needed to find out hypertension problems.

### 3.3. CVD and SCORE Risk Charts

The analysis of blood pressure levels and other risk factors can be used to determine CVD. In this sense, the SCORE method is able to estimate the 10-year risk of a first fatal atherosclerotic event, whether heart attack, stroke, aneurysm of the aorta, or other kind of CVD. Besides, SCORE charts provide a set of variables that identify the inputs to the reasoning engine. These ones are shown in Table 1 and below.

On the other hand, the outputs of the reasoning module from the previous variables have been grouped as follows.

- ***Very High***. If a user presents a risk of 15% and over.
- ***High***. If the risk is in the range 10%–14%.
- ***Mid High***. User presents a risk from 5% to 9%.
- ***Mid***. User presents a risk from 3% to 4%.
- ***Mid Low***. If the risk is 2%.
- ***Low***. If the risk presented corresponds to 1%.
- ***None***. No risk is presented.

In addition, recommendations can be offered to the physician through a specific mobile application. These recommendations can be generated from the reasoning outputs and other clinical factors (including chronic diseases such as diabetes, and other pathologies). However, our initial prototype determines the CVD risk from the patient profile and an average of latest blood pressure measures.

### 3.4. Reasoning Module

The reasoning module calculates the CVD risk associated to a patient and has the ability to create patient recommendations to decrease his CVD risk. The reasoning engine uses the OWL API [22] to load the patient ontology and the SWRL rules and Pellet [23] reasoner to perform the reasoning task. In our case, more than 250 rules have been described according to SCORE charts.

**Figure 2.**
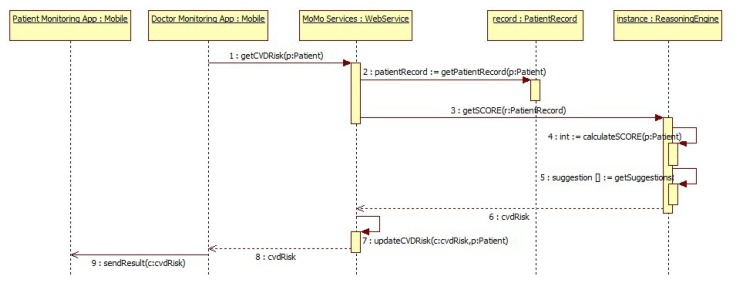
Sequence diagram: CVD Risk calculation.

SWRL rules are divided in two parts: the antecedent and the consequent. The antecedent describes the conditions that must be fulfilled to infer the consequent assumptions; in this case, the antecedents are the patient's health condition. The consequences are the patient's CVD risk and a set of recommendation adapted to him. Final results are sent to a medical mobile application through the corresponding web services. Figure 2 shows the sequence diagram from this process and Table 2 shows an example of a complete SWRL rule according to the specific output provided by SCORE.

### 3.5. Recommendation Mechanisms

Reasoning techniques allow us to determine what recommendations should be given to the user not only based on information proactively inferred through reasoning techniques according to their current situation (*i.e.*, current physical activity) and elements of his surrounding (*i.e.*, the weather). Without a CDSS patients receive general recommendations from physicians based on their health state. Physician's recommendations suggest to perform moderate or vigorous exercise, control diet by eating more vegetables and fruits, limiting the unhealthy fats and selecting whole grains, *etc.* To integrate those recommendations into patients' daily life, it is necessary to include more complex mechanisms. This is specially required whenever patients suffer other diseases apart from CVD such as kidney failure or diabetes.

**Figure 3.**
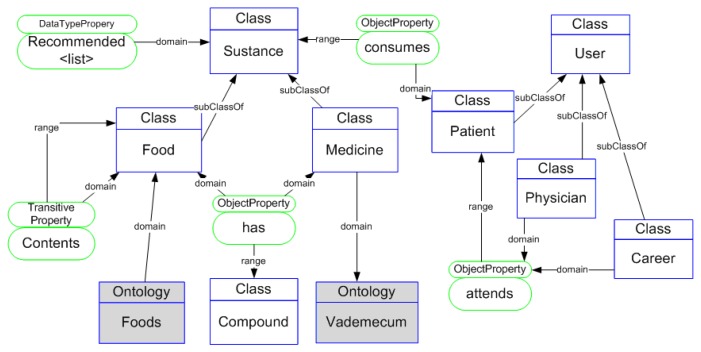
Ontological concepts and properties involved in the example of axiomatic inference.

As an application example, let us suppose that a patient has a moderate CVD risk and suffers kidney failure. In the system there is an ontology that models medicines and dishes. Medicines and dishes may have compounds like active or chemical principles. Any food could be allowed, partially acceptable or forbidden for the patient. Figure 3 shows the relevant context submodel of this example. When we need to decide which dishes a patient can take, the system checks if the food composition is appropriate for a given patient. As example, we can assume that the patient is going to eat a herring. The knowledge base does not include the composition of herrings but knows that herrings belong to the family clupeidae, as pilchards do. Knowing that “herrings” is a member of the class Clupeidea, the system infers that herrings can have potassium, like pilchards, a dangerous component to people with kidney failure. In a similar way, the system could infer that the patient is taking a dangerous food if any of its components is dangerous, using the transitive property < *contents* >.

In general, reasoning techniques enable the definition of behaviour rules, improving the information quality and the reasoning power. Consequently, these reasoning techniques allow us to determine what recommendation should be shown to the user, not only based on the explicit data about user situation or user health status but also information proactively inferred through behavioural rules. This dynamic behaviour is achieved again using SWRL. In the following, we describe several examples of rules that can launch recommendations to patients. Listing 1 represents the fact that the patient is a children with moderate CDV Risk, *i.e.*, SCORE bigger than 3 (lines 1 and 2), follows a daily diet (line 3) should consume less fat than the 30% of total calories, and at maximum the 10% of saturated fats [24]. This rule launches a recommendation whenever the patient exceeds that level.


**Listing 1.** SWRL rule to detect excessive fats in the patient diet.
1:talismanPlus:Patient (?p) Λ talismanPlus: hasCVDRisk (?p, ?cvd) Λ talismanPlus:age (?p,?age; Λ2:swrlb:stringEqualsIgnoreCase(?cvd, “Hig”) Λ swrlb: swrlb:lessThan (?age, 45) Λ3:talismanPlus:Diet (?d) Λ talismanPlus:dailyDiet (?p, ?d) Λ4:talismanPlus:Fat (?d, ?fat) Λ swrlb:greatherThan (?fat, 0.30) Λ5:talismanPlus:SFat (?d, ?sfat) Λ swrlb:greatherThan (?sfat, 0.10) Λ **→** talismanPlus:recomm (?u,“Be careful with current Fat it will increase your CVD risk”)

Listing 2 describes a rule to show a reminder whenever user forgets taking a medicine.


**Listing 2.** SWRL rule to remind to take the medicines.
talismanPlus:Patient(?x) Λ talisman-core:isDoing(?x, ?activity) Λtalisman-core:wasAt(?activity, ?time) Λj.0:medicalTreatment(?x, ?treatment) Λ j.0:lastDose(?treatment, ?last) Λj.0:every(?treatment, ?every) Λswrlb:subtract(?difference, ?time, ?last) Λ swrlb:greaterThanOrEqual(?difference, ?every) Λj.0:medicamentName(?treatment, ?uri) Λ swrlb:stringConcat(?command, “Period treatment”, ?uri) Λswrlb:greaterThan(?every, 0)**→** p1:messages(?x, ?command)

Finally, listing 3 represents the rules to check whether user exceed the screen time, that is, the time of sedentary behaviour.


**Listing 3**. SWRL rule to remind to interrupt a detected sedentary behaviour.
talismanPlus:Patient (?u) Λ talisman-core:isDoing(?u, ?pa) Λ talismanPlus:PhyActivity(?pa) Λtalisman-core:wasAt(?activity, ?time; Λ talismanPlus:Time(talismanPlus:Time,?t) Λswrlb:subtract(?difference, ?t, ?time) Λ swrlb:greaterThanOrEqual (?time, 60)**→** talismanPlus:recomm (?u, “We encourage you to perform an activity”)

All these examples were designed with the assumption of a complete knowledge of the context, that is, the users or sensors in the surroundings include into the system the required information regarding daily activities such as medicines taken, diet and physical activities.

### 3.6. System Deployment

**Figure 4.**
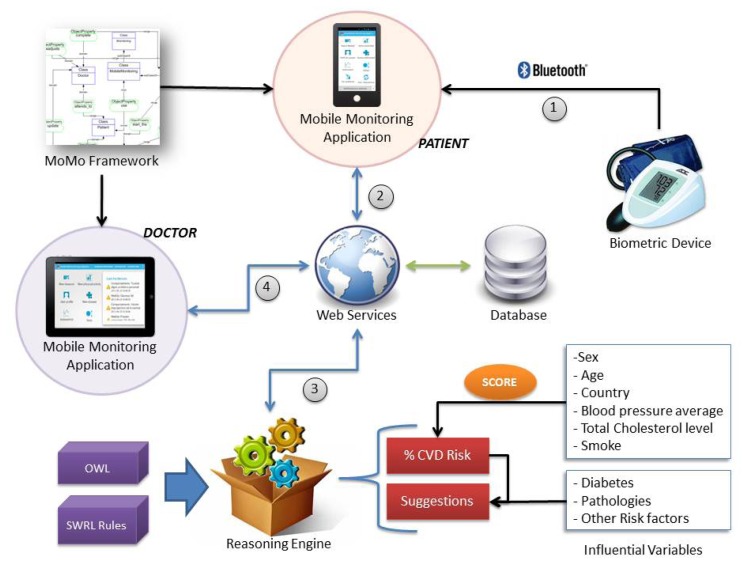
System Overview.

The mobile monitoring system and the reasoning module have been integrated in a single architecture with the following elements:

- Mobile Monitoring Applications. Two Android (http:www.android.com) applications have been implemented to perform the monitoring tasks. The first mobile application allows the patients to monitor their vital signs such as blood pressure as explained in this paper(see Figure 4, step 1). The second application allows physicians to obtain the monitoring results of the patients at real time, in this case, regarding to their cardiovascular disease risk. Both include information and charts related to the monitored tasks
- MoMo Framework. The mobile applications have been developed by using the MoMo framework. Thus, we can extend the functionality of the applications or their monitoring requirements in the future among other features.
- Biometric device. The biometric device allows us to collect measures from specific vital signs. In this case, blood pressure measures are sent to the mobile phone via Bluetooth as we mentioned in Section 3.2. This type of device provides us an open protocol to establish the communication and send blood pressure values via Bluetooth.
- Storage System. Both patient profile factors and final results provided by our system are stored in a server database. This information is accessible through web services (see Figure 4, step 2, 3 and 4).
- Web services. All the transactions between the mobile applications, the storage system and the reasoning engine are mediated by web services. These represent the core of our system and the only communication method that manages requests from the end users.
- Reasoning engine. This element is also known as reasoning module and it is responsible for calculating the CVD risk from the OWL ontology and the associated SWRL rules.

Currently, the reasoning task works with SCORE method. Additionally, this module allows to create new rules for providing recommendations and suggestions to patients thanks to the CVD risk value, in combination with other influential variables from the patient record. In Section 3.5 were shown several examples of these types of rules. The whole system provides a continuous monitoring having a direct contact with the patient and these suggestions may also be completed and enriched by doctor criteria.

## 4. Evaluation and Benchmark Tests

In this section we present the results of the user evaluation and the benchmark tests carried out.

### 4.1. Usability and User Satisfaction Evaluation

**Figure 5.**
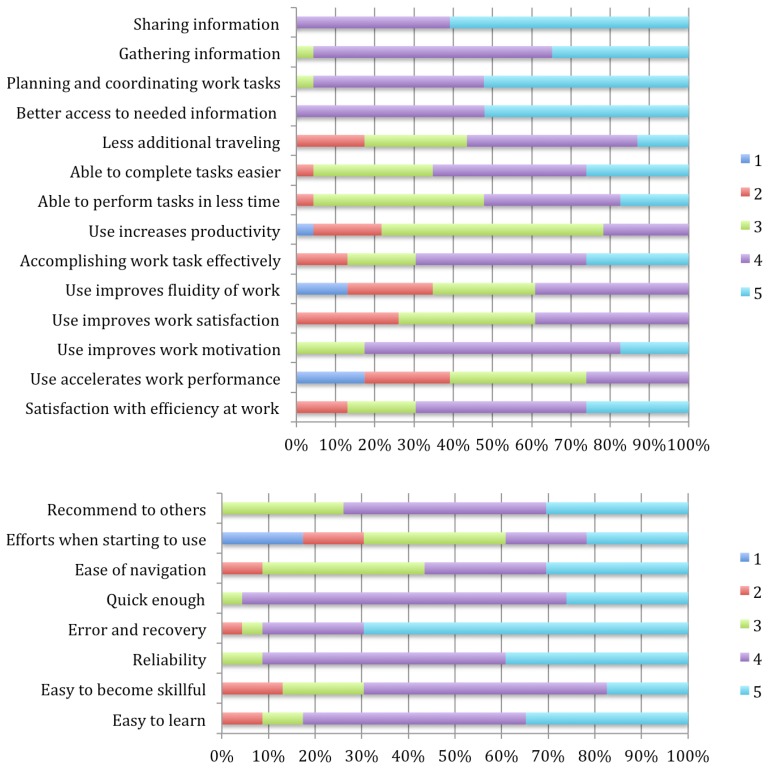
Summarized results about the use of the applications to monitoring patients with CVD. According to the ease of use, patients feel that the application is easy to use because the interaction is very simple and short and the mobile application responds mostly automatically.

We have evaluated the mobile application through interviews and user studies to understand how a patient uses the mobile application to control their biometrical parameters, for example, blood pressure. Additionally, we have checked whether the visualization mechanisms and user interfaces are suitable for the end-user. Twenty-three users (fourteen men, nine women) have participated in the experiment over a period of four days. The experiments were incorporated into their daily activities to simulate actual situations. The amount of time that each user tested our prototypes was 40 minutes per day on average. The population included ten retired users and thirteen active users with different professional profiles, all between the ages of 35 and 72. This evaluation (see Figure 5) is focused on user experience. These items are a subset of the MoBiS-Q questionnaire [25] and we have applied a Likert-type scale to evaluate the validity of each item, where 5 is the highest rating meaning “fully satisfactory” and 1 is the lowest rate meaning “not satisfactory at all”.

First, the patient measures his/her blood pressure. Once the blood pressure is read, the pressure meter displays the patient levels and sends these values to the mobile device. Then, the patient can graphically visualize the measures trends during last days. The user can also introduce and visualize information about their daily activities such as diet, medicines and physical activities. The goals of this evaluation are to know if the patient accepts this solution and to evaluate the functionality of the mobile monitoring application. This evaluation was applied to patients with hypertension, where the mobile device is used to monitor their blood pressure levels in conjunction with annotations about their diet and activities. In general, the users gave high ratings to most of items. Specifically, the mode was 4 out of 5. Since we are performing a Likert based experiment, we will report mode values instead of average values. Obtained mode values reported a high satisfaction level, especially in the first four items. They gave lower ratings to issues related to the way to input information; thus, it could be improved. We plan to adapt our previous contributions on physical activity recognition through accelerometers [26] and touching interaction based on Near Field Communication [27] to improve and automate those aspects.

In general, this statistical analysis helped us to estimate the application suitability for the user. Nevertheless, a better evaluation is necessary regarding the number of participants and statistical techniques. We will explain these issues in more detail in the conclusions section.

**Figure 6.**
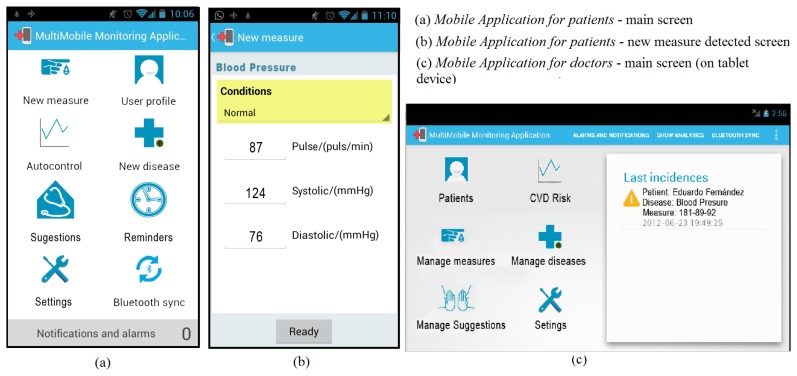
Mobile application graphical user interface.

In summary and as Figure 5 shows, the aspects evaluated were the following:

- Usability of the application. Usability has been evaluated regarding to visual and functional aspects of the graphical user interface (see Figure 6). The visual aspect belongs to the ability to use the application according to the interfaces developed or how friendly the interfaces are for patients.
- Assessment of the application in comparison with the handwriting of the blood pressure levels and annotation of daily activities. We could check if the application has integrated the same functionalities of handwritten annotations and if the mobile application could improve these tasks.A 62% of users indicated that, thought the application, they had a better experience than the handwritten annotations.
- Response time of the application. This aspect evaluates the required time to provide answers for medical surveillance after the biometrical levels of the patient are obtained. After this aspect was evaluated, eighteen users marked a rating of good or very good (4–5), while rest of users rated this issue as moderate (3).

### 4.2. Measuring the Performance of CVD Risk Reasoning Services

An application should be intuitive and easy to use and also should have a good performance and a satisfactory time response. A poor performance degrades the user engagement and the experience of an application. To test the behaviour of our system we have employed the JMeter (http://jmeter.apache.org/) tool and measured its performance with heavy load. This tool offers a straightforward method to simulate the load on a server, defining the number of requests, the ramp-up period and how many times the experiment must be repeated. The ramp-up period determines the interval time that JMeter has to wait before starting each new user's request. Due to the intrinsic nature of this system, we propose 100 physicians as the maximum number of concurrent users, since it is very uncommon to have more than 100 physician requests concurrently. Therefore, we tested the system with 10, 50 and 100 simulated requests. According to [28] the average face-to-face patient care is 10.7 minutes. This study reinforces the issue that less than 7 requests need to be handled per hour.

Our test plan is composed by nine experiments, which can be divided in three groups depending on its ramp-up period:

- 0 seconds ramp-up period.
- 5 seconds ramp-up period.
- 10 seconds ramp-up period.

**Figure 7.**
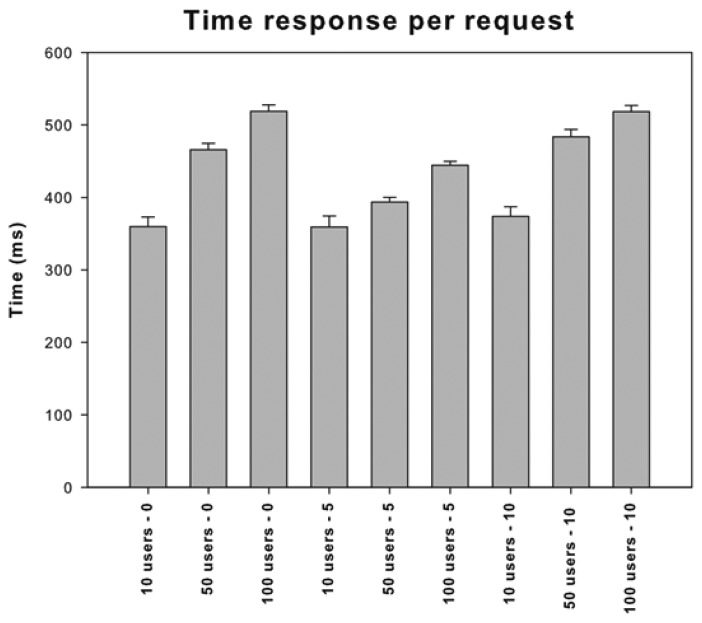
Mean and standard error of time queries for different combination of users.

**Figure 8.**
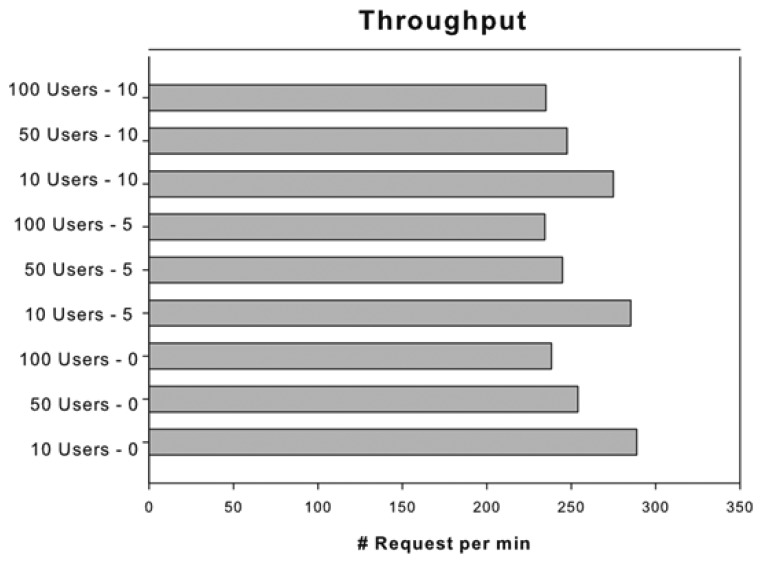
Obtained throughput results for different combination of users.

In each group we measure the server load with 10, 50 and 100 simulated users' requests and repeat them 50 times. We generated one thousand random requests with different users' data, which were also randomly chosen from the JMeter test plan. CVD risk web services were deployed in an Apache Tomcat 7.0.29 server running on an Acer Aspire 4830TG laptop with an Intel Core i5-2410M and 8 GB of RAM. The operating system is Ubuntu 11.10 for 64 bits, a distribution of GNU/Linux, which runs the Java 1.6.0 26 version. The JMeter data collection was executed on a different laptop and both computers were connected to the same local network. As Figure 7 shows, the mean response time is below 520 milliseconds for all the tests. The best response time for 50 and 100 users was reported with the “5 seconds ramp-up period” and the best response time for 10 users was in the “0 seconds ramp-up period” group. Figure 8 shows that the server can attend more than 200 requests per minute and almost 300 request per minute.

To summarize, we have shown that the total request and execution time is not very high and the system could be deployed in nursing homes, community health centres and hospitals in an appropriate way. Besides, using real server machines instead of commodity laptop could improve the results and cut down the response time.

## 5. Conclusions

In this work, we have presented a system that uses a mobile phone to monitor the blood pressure of a patient and a reasoning engine to calculate the CVD risk applying the SCORE method over the blood pressure values and the other clinical factors. Additionally, the system has been extended with new SWRL rules to create specific recommendations according to the patient profile and situation. As future work, we propose the integration of these new rules to check the body mass index and glucose levels in combination with the calculated CVD risk. We also believe that it is possible to provide an appropriate treatment and medication considering these factors.

The solution presented in this paper is focused on adults over 45 years as SCORE proposed and it is only valid for European regions. We believe that the generalization of this system taking into account other methods (see Section 2) and age ranges could meet the needs of other population groups like children. For that, new rules and variables must be considered as part of the ontology. The use of biometric devices is determined for their communication protocols and interoperability features. We need devices with open or standardized protocols as proposed by the Continua Health Alliance from the ISO/IEE 11073 group. (http://www.iso.org/iso/search.htm?qt=11073&searchSubmit=Search&sort=rel&type=simple&published=true). However, currently most existing devices do not integrate these features.

Additionally, the system depends on the user actions, *i.e.*, the patient must take the blood pressure himself to obtain the corresponding measures. In this sense, notifications and reminders can facilitate this fact. If we consider to deploy the system in a real scenario such as hospital or to integrate it in other systems, we must apply interoperability standards like Health Level Seven (HL7) (http://www.hl7.org/) to manage health information in a convenient fashion.

We carried out several experiments and they have shown that our system fulfils users' expectations and provides a good performance. In particular, users have expressed a high level of acceptance of the manner in which they can check and monitor their disease. In general, the usability of the mobile application obtained a rate of acceptance of 69%. This rate, from a general point of view, is reasonable. The majority of users were elders and/or people that rarely used assistive technology. The low-rate of acceptance of assistive technologies in elder people has been analysed in [29,30], so we consider our rate promising and it encourages us to continue improving this work. The visual representation of the information and their integration into the user interfaces has been also positively evaluated. However, the statistical analysis of usability is not totally suitable, thus we plan to perform new tests including a higher number of participants and using non-parametric techniques and tests to avoid some statistical inconsistencies regarding to distribution data.

Regarding the performance of the reasoning services, we have also shown that the total requests and execution time is acceptable and the system could be deployed in nursing homes, community health centres and hospitals in an appropriate way.

## Figures and Tables

**Table 1.** Input Variables.

**Variable**	**Description**	**Type**	**Range**
Sex	Gender of the person	Binary	Male or Female
Age	Age of the person	Discrete	[40,50,55,60,65]
Smoker	Indicates if the person smokes	Binary	True or False
Cholesterol	Cholesterol level (mmol/L)	Double	[4,5,6,7,8]
Blood Pressure	Average of Systolic Blood Pressure (mmHg)	Discrete	[120,140,160,180)
High Risk Country	Indicates if the person lives in a high risk country (view the list of the countries below)	Binary	True or False

*List of Low Risk Countries:* Andorra, Austria, Belgium, Cyprus, Denmark, Finland, France, Germany, Greece, Iceland, Ireland, Israel, Italy, Luxembourg, Malta, Monaco, The Netherlands, Norway, Portugal, San Marino, Slovenia, Spain, Sweden ILAR, Switzerland, United Kingdom; *List of High Risk Countries*: Other European countries such as Armenia, Azerbijan, Belarus, Bulgaria, Georgia, Kazakhstan, Latvia, Lithuania, Macedonia FYR, Moldova and Ukraine, among others.

**Table 2.** Example of SWRL Rule based on SCORE for a 40-years-old non-smoker woman who lives in a low CVD risk country, whose systolic blood pressure is between 120 and 160 mmHg and whose cholesterol is between 4 and 6 mmol/L.

**Antecedents**	

*Conditions*	*SWRL Translation*

Pick up an individual who is a Patient	talismanPlus:Patient(?patient) Λ

Where does she live?	talismanPlus:livesIn(?patient,?country)Λ
	talismanPlus:LowCVDRiskCountry(?country)Λ

Is she a female?	talismanPlus:isMale(?patient,?isMale)Λ
	sqwrl:equal(?isMale,false)Λ

How old is she?	talismanPlus:isYearsOld(?patient,?years)Λ
	swrlb:greaterThanOrEqual(?years,40)Λ
	swrlb:lessThan(?years,50)Λ

Does she smoke?	talismanPlus:isSmoker(?patient,?smoke)Λ
	sqwrl:equal(?smoke,false)Λ

Obtain her record	talismanPlus:hasRecord(?patient,?history)Λ

Check her systolic blood pressure	talismanPlus:hasTest(?history,?systolic)Λ
	talismanPlus:SystolicBloodPressureAvgTest(?systolic)Λ
	talismanPlus:hasSystolicBloodPressure(?systolic, ?systolicMeasure)Λ
	swrlb:greaterThanOrEqual(?systolicMeasure,120)Λ
	swrlb:lessThan(?systolicMeasure,160)Λ

Check cholesterol	talismanPlus:hasTest(?history,?cholesterol)Λ
	talismanPlus:CholesterolTest(?cholesterol)Λ
	talismanPlus:hasCholesterol(?cholesterol, ?cholesterolMeasure)Λ
	swrlb:greaterThanOrEqual(?cholesterolMeasure,4)Λ
	swrlb:lessThan(?cholesterolMeasure,6)

**Consequent**	

*Action*	*SWRL translation*

small Set her CVD risk	**→** talismanPlus:hasCVDRisk(?patient,“none”)
